# Immune imbalance underlying depressive symptoms in COPD patients: a study based on BDNF, PD-1, MMP-9, and inflammatory cytokines

**DOI:** 10.3389/fmed.2025.1606630

**Published:** 2025-08-01

**Authors:** Shanshan Liu, Jian Dong, Weiqi Huang, Jing Yan

**Affiliations:** ^1^Department of Neurology, Union Jiangbei Hospital, Huazhong University of Science and Technology, Wuhan, China; ^2^Department of Respiratory and Critical Care Medicine, Union Jiangbei Hospital, Huazhong University of Science and Technology, Wuhan, China

**Keywords:** chronic obstructive pulmonary disease (COPD), immune biomarkers, brain-derived neurotrophic factor (BDNF), programmed cell death protein 1 (PD-1), matrix metalloproteinase-9 (MMP9), inflammatory factors

## Abstract

**Objective:**

Chronic obstructive pulmonary disease (COPD) is frequently accompanied by a high prevalence of depressive symptoms, particularly during acute exacerbations (AECOPD). However, the immunoinflammatory mechanisms underlying AECOPD-associated depression remain poorly elucidated. This study aimed to investigate the potential roles of brain-derived neurotrophic factor (BDNF), programmed cell death protein 1 (PD-1), matrix metalloproteinase-9 (MMP-9), and key inflammatory cytokines—interleukin-1β (IL-1β), interleukin-10 (IL-10), and tumor necrosis factor-*α* (TNF-α)—in mediating depressive symptoms among hospitalized AECOPD patients. The findings aim to clarify the contribution of immune dysregulation to the development of depression in this population.

**Methods:**

A total of 140 patients hospitalized for AECOPD and 50 age- and sex-matched healthy controls were recruited. Patients were stratified into depressive (HAMD ≥ 17) and non-depressive (HAMD < 17) groups based on the Hamilton Depression Rating Scale. Following a 90-day follow-up, patients were further categorized into stable and recurrent exacerbation subgroups. Serum levels of BDNF, PD-1, MMP-9, IL-1β, IL-10, and TNF-α were measured using peripheral blood samples. Intergroup comparisons were conducted, and correlations between biomarker levels and depression severity were analyzed. Multivariate logistic regression was performed to identify independent risk and protective factors for depressive symptoms.

**Results:**

Compared with healthy controls, AECOPD patients showed significantly reduced BDNF levels (0.225 vs. 0.575, *p* < 0.001) and elevated PD-1 levels (0.865 vs. 0.255, *p* < 0.001). Within the patient cohort, individuals with depressive symptoms exhibited lower BDNF (0.13 vs. 0.24, *p* < 0.001) and higher PD-1 expression (0.89 vs. 0.78, *p* < 0.001) than those without depression. Multivariate analysis identified PD-1 (OR = 3.32) and MMP-9 (OR = 2.18) as independent risk factors for depression, while IL-10 (OR = 0.62) and BDNF (OR = 0.12) emerged as protective factors. Smoking status was also recognized as a modifiable risk factor (OR = 1.73).

**Conclusion:**

Depressive symptoms in AECOPD patients appear to be driven by a multifaceted interplay involving neuroinflammation (characterized by BDNF reduction and elevated IL-1β/TNF-α), immune dysregulation (marked by PD-1 upregulation and IL-10 suppression), and extracellular matrix remodeling (via increased MMP-9). Modulation of the PD-1/MMP-9 axis may offer a novel therapeutic strategy, while smoking cessation could potentiate BDNF-related neuroprotective effects.

## 1 Introduction

Chronic obstructive pulmonary disease (COPD) is a progressive respiratory disorder characterized by persistent airflow limitation (1), and remains a leading global cause of morbidity and mortality (2). Beyond respiratory impairment, COPD frequently coexists with a spectrum of systemic comorbidities, among which depressive symptoms are particularly prevalent, with a reported prevalence exceeding 40% (3, 4). Depression in COPD is associated with reduced treatment adherence, increased hospitalization rates, and poorer overall prognosis. Despite its clinical significance, the underlying immunobiological mechanisms connecting COPD and depression remain inadequately understood.

In recent years, immune dysregulation and chronic inflammation have been increasingly recognized as pivotal contributors to the pathogenesis of both COPD and depression (5). COPD-induced systemic inflammation may influence central nervous system (CNS) function through neuroimmune pathways, thereby elevating the risk of depressive disorders (6). Inflammatory mediators originating from the periphery may traverse a compromised blood–brain barrier (BBB) or act via the vagus nerve and microglial priming, thus initiating neuroinflammatory cascades that disrupt affective regulation. These processes are likely exacerbated during acute exacerbations of COPD (AECOPD), when systemic inflammatory activation intensifies, potentially increasing the susceptibility to depressive symptoms. To investigate these mechanisms, this study focuses on a panel of neuroimmune biomarkers with established or putative relevance to COPD and depression. Brain-derived neurotrophic factor (BDNF) is a key neurotrophin essential for synaptic plasticity, neurogenesis, and mood regulation. Decreased BDNF has been repeatedly linked to depression, and inflammatory states such as COPD may suppress its expression (7, 8). Programmed cell death protein 1 (PD-1) is an immune checkpoint receptor that modulates T-cell activity and prevents immune overactivation. Dysregulated PD-1 signaling may contribute to chronic inflammation and immune exhaustion, potentially influencing central neuroimmune interactions and affective dysfunction (9). Meanwhile, matrix metalloproteinase-9 (MMP-9) facilitates extracellular matrix degradation and has been implicated in BBB disruption, peripheral-to-central cytokine trafficking, and depression-related neuroinflammatory processes (10, 11). Among cytokines, interleukin-1β (IL-1β) and tumor necrosis factor-α (TNF-α) are central to both COPD pathophysiology and neuroinflammation. They promote microglial activation and alter monoaminergic neurotransmission. These cytokines have been linked to both airway inflammation and major depressive disorder by activating microglial cells and disrupting neurotransmitter pathways (12, 13). Conversely, interleukin-10 (IL-10) is an anti-inflammatory cytokine that counteracts immune overactivation. Reduced IL-10 levels, commonly seen in depression, may weaken immune regulation and amplify the inflammatory burden in COPD (14). While previous studies have investigated some of these biomarkers in isolation, a comprehensive evaluation of their collective roles in the immunopathogenesis of depression during AECOPD episodes remains lacking. The present study aims to address this gap.

Therefore, the objectives of this study are: (1) To compare serum levels of BDNF, PD-1, MMP-9, IL-1β, IL-10, and TNF-α between AECOPD patients and healthy controls; (2) To investigate their associations with depressive symptom severity (as assessed by the Hamilton Depression Rating Scale); (3) To explore the hypothesis that immune imbalance—including neurotrophic impairment, pro-inflammatory activation, and reduced anti-inflammatory signaling—constitutes a central mechanism underlying depression in COPD.

Through this investigation, we seek to elucidate immune-driven mechanisms underlying depression during the acute phase of COPD and to identify candidate biomarkers for targeted intervention.

## 2 Materials and methods

### 2.1 Study population

This prospective cross-sectional study designed to collect clinical data, blood samples, and depression assessments from patients with COPD. A retrospective approach was employed to recruit patients hospitalized for acute exacerbation of chronic obstructive pulmonary disease (AECOPD) at Jiangbei Hospital, Huazhong University of Science and Technology, between January 2023 and June 2024.

Additionally, 50 healthy volunteers were recruited during the same period as controls.

### 2.2 Ethics statement

This study was approved by the Institutional Review Board (IRB) of Union Jiangbei Hospital, Huazhong University of Science and Technology (Approval No.: LLSC2022042005), and conducted in full accordance with the Declaration of Helsinki (2013 revision) (15).

All participants or their legally authorized representatives provided written informed consent before inclusion. For elderly participants with mild cognitive impairment not due to organic mental disorders, consent was obtained from their legal guardians.

All personal data were anonymized and processed confidentially. The study protocol complied with all relevant institutional and national ethical guidelines governing research involving human participants.

### 2.3 Inclusion and exclusion criteria

#### 2.3.1 Inclusion criteria

Eligible participants met the following criteria:

1. Aged 18 years or older;
2. Confirmed diagnosis of COPD based on spirometry and imaging findings, consistent with national and international diagnostic guidelines (16, 17);
3. Hospital admission within 48 h of acute exacerbation onset, and completion of standardized AECOPD treatment prior to discharge;
4. No prior diagnosis of depression or use of antidepressant medications;
5. Complete clinical records available and willingness to participate in a 90-day follow-up.

#### 2.3.2 Exclusion criteria

Patients were excluded if they met any of the following:

1. Comorbid conditions that may confound the study, such as active pulmonary infections, malignancies, or autoimmune diseases;
2. Discontinuation of treatment during hospitalization;
3. Personal or family history of psychiatric disorders, or diagnosis of organic mental illness;
4. Underwent surgical procedures within 15 days before admission;
5. Pregnancy or breastfeeding at the time of enrollment.

### 2.4 Study grouping

At baseline, patients were stratified according to depression severity, and subsequently reclassified at the 90-day follow-up based on their exacerbation status. Depression status was determined using the Hamilton Depression Rating Scale (HAM-D), with a score ≥17 used to define clinically significant depression, in accordance with psychosomatic medicine guidelines (18, 19):

Depression group: HAM-D score ≥17.
Non-depression group: HAM-D score <17.

At the 90-day follow-up, patients were further categorized into exacerbation and stable groups based on objective criteria for acute exacerbation of COPD (AECOPD) events (20).

·Exacerbation group: Patients who met ≥1 major criterion or ≥2 minor criteria:

- Major criteria (requiring hospitalization):
- Acute respiratory failure (PaO₂ < 60 mmHg ± PaCO₂ > 45 mmHg).
- New-onset cyanosis/peripheral edema.
- Minor criteria (managed in outpatient setting):
- Sustained worsening of dyspnea (mMRC increase ≥1 grade for ≥48 h).
- Increased sputum volume + purulence (Anthonisen Type I).
- CAT score increase ≥4 points from baseline.

Stable group: patients who did not meet any exacerbation criteria and fulfilled all of the following:

- No respiratory-related emergency visits/hospitalizations.
- No requirement for systemic corticosteroids (prednisone ≥20 mg/day) or antibiotics.
- CAT score fluctuation ≤3 points from baseline.

Validation: All exacerbation events were adjudicated by two board-certified pulmonologists who were blinded to biomarker data (*κ* = 0.91). The COPD Assessment Test (CAT) (21) and the modified Medical Research Council (mMRC) Dyspnea Scale (22), were used to assess symptom burden, psychological status, and dyspnea severity.

Note: Comorbidities such as chronic kidney disease and cardiovascular diseases were not excluded to reflect the real-world clinical profile of AECOPD patients.

### 2.5 Data collection

Baseline demographic and clinical data were collected upon enrollment, including age (reported as median and interquartile range), sex, and smoking status. These variables were analyzed for potential associations with clinical outcomes.

For AECOPD patients, fasting venous blood samples were obtained at two defined time points:

1. Within 24 h of hospital admission, prior to initiation of systemic treatment.
2. At the 90-day post-discharge outpatient follow-up visit.

For healthy controls, a single fasting blood sample was collected during a routine morning health examination.

All blood draws were conducted between 6:00 and 8:00 AM following an overnight fast. Samples were centrifuged at 3,000 × g for 10 min and immediately stored at −80°C to preserve biomarker integrity. A standardized pre-analytical protocol was used across all subjects to minimize variability.

Serum levels of BDNF, PD-1, MMP-9, and cytokines (IL-1β, IL-6, IL-10, IL-18, TNF-α) were quantified using enzyme-linked immunosorbent assays (ELISA) performed in a blinded laboratory setting. All measurements were conducted in duplicate using validated commercial kits with high inter-assay reproducibility.

BDNF antibody: Abcam, No. 66220-1-IG.

PD-1 antibody: Proteintech, No. 28205-1-AP.

Cytokines: Abcam, No. 10375-2-AP (shared catalog for IL-1β, IL-10, TNF-α).

### 2.6 Statistical analysis

All statistical analyses were performed using R software (version 4.2. 1) and the *stats* package. Data normality was assessed prior to analysis. Continuous variables were expressed as mean ± standard deviation (SD) or median with interquartile range (IQR), depending on distribution. Between-group comparisons were conducted as follows:

For normally distributed data with equal variance: independent *t*-testFor non-normally distributed data: non-parametric tests such as the Wilcoxon rank-sum test or Kruskal-Wallis testFor categorical variables: Chi-square test, A two-tailed *P* value <0.05 was considered statistically significant.

## 3 Results

### 3.1 Baseline characteristics

Baseline characteristics of the study population are summarized in Table 1. There were no statistically significant differences between the AECOPD group and healthy controls with respect to age (*p* = 0.858), sex distribution (*p* = 0.873), or smoking status (*p* = 0.647). However, patients in the AECOPD group exhibited significantly lower pulmonary function (FEV₁% predicted: 46% vs. 98%, *p* < 0.001), higher symptom burden (CAT score: 21 vs. 8, *p* < 0.001; mMRC: 2 vs. 0, *p* < 0.001), and elevated depressive symptom scores (HAM-D: 16 vs. 4, *p* < 0.001).

**Table 1:** Baseline characteristics.

Characteristics (*n*)	AECOPD (*n* = 140)	Control (*n* = 50)	*p* value
Age, median, median (IQR)	70 (66, 76)	73 (59, 75)	0.858
Male, *n* (%)	97 (69.30%)	41 (82.00%)	0.873
Smoking Status, *n* (%)	89 (63.6%)	34 (68.00%)	0.647
FEV1%pred, median (IQR)	46 (31, 63)	98 (92, 105)	< 0.001
FVC%pred, median (IQR)	84 (71.98)	102 (95,108)	< 0.001
FEV1/FVC%, median (IQR)	46 (39, 62)	74 (70, 77)	< 0.001
CAT score, median (IQR)	21 (16.27)	8(8,9)	< 0.001
mMRC, median (IQR)	2 (1,3)	0 (0,0)	< 0.001
Cerebrovascular Disease, *n* (%)	62 (44.30%)	0 (0%)	< 0.001
Hypertension, *n* (%)	41 (29.30%)	0 (0%)	< 0.001
Coronary Artery Disease, *n* (%)	35 (25.00%)	0 (0%)	< 0.001
Diabetes, *n* (%)	5 (3.60%)	0 (0%)	0.505
COPD with Sleep Apnea Syndrome, *n* (%)	10 (7.10%)	0 (0%)	0.178
Hamilton Depression Scale, median (IQR)	16 (12, 21)	4 (2, 7)	< 0.001
Number of exacerbations in past 12 months, *n* (%)			
0 times	87 (62.14%)	**–**	NA
1 times	31 (22.14%)	**–**	NA
≥2 times	22 (15.71%)	**–**	NA
GOLD stage, *n* (%)			
1	21 (15.00%)	**–**	NA
2	45 (32.14%)	**–**	NA
3	56 (40.00%)	**–**	NA
4	18 (12.86%)	**–**	NA
Systemic corticosteroids during hospitalization, *n* (%)	104 (74.29%)	**–**	NA
Antibiotic use during hospitalization, *n* (%)	115 (82.14%)	**–**	NA
Theophylline use during hospitalization, *n* (%)	137 (97.86%)	**–**	NA
Inhaled corticosteroids (ICS) prior to admission, *n* (%)	63 (45.00%)	**–**	NA
Inhaled bronchodilator, *n* (%)	58 (41.43%)	**–**	NA
Oxygen therapy, *n* (%)	94 (67.14%)	**–**	NA
Noninvasive ventilation assisted treatment, *n* (%)	24 (17.14%)	**–**	NA
Type II respiratory failure	28 (20.00%)	**–**	NA

Data are expressed as median (IQR) or *n* (%). *p*-values were calculated using Mann–Whitney U test or *χ*^2^ test. No effect sizes are reported as this table serves a descriptive purpose. AECOPD, acute exacerbation of chronic obstructive pulmonary disease; CAT, COPD Assessment Test; mMRC, modified Medical Research Council dyspnea scale; HAM-D, Hamilton Depression Scale; FEV₁, forced expiratory volume in one second; FVC, forced vital capacity; ICS, inhaled corticosteroids; GOLD, Global Initiative for Chronic Obstructive Lung Disease; IQR, interquartile range; NA, not applicable.

Furthermore, the AECOPD group showed a markedly higher prevalence of vascular comorbidities, including:

Cerebrovascular disease (44.3% vs. 0%, *p* < 0.001),
Hypertension (29.3% vs. 0%, *p* < 0.001), and
Coronary artery disease (25.0% vs. 0%, *p* < 0.001).

There were no significant between-group differences in the rates of diabetes mellitus (*p* = 0.505), or COPD–overlap with obstructive sleep apnea syndrome (*p* = 0.178).

Among AECOPD patients, over one-third had experienced at least one exacerbation in the past year. Most were classified as GOLD stage 2 or 3, indicating moderate to severe airflow limitation. During hospitalization, the majority received systemic corticosteroids (74.3%), antibiotics (82.1%), and theophylline (97.9%). Pre-admission therapy included inhaled corticosteroids (45.0%) and bronchodilators (41.4%). Oxygen therapy was required in 67.1% of cases, and 17.1% received noninvasive ventilation. Type II respiratory failure occurred in 20.0% of patients, reflecting the severity of illness in this cohort.

### 3.2 Significant differences in all assessed biomarkers between AECOPD patients and healthy controls

Patients with acute exacerbation of chronic obstructive pulmonary disease (AECOPD) exhibited significant alterations across all assessed peripheral biomarkers compared to healthy controls (see Table 2).

**Table 2:** Biomarker level comparison between AECOPD patients and healthy controls.

Biomarker	AECOPD group (*n* = 140, median IQR)	Healthy control group (*n* = 50, median IQR)	Cohen’s d	*p* value
BDNF	0.23 (0.15, 0.28)	0.58 (0.46, 0.64)	−2.94	< 0.001
PD -1	0.87 (0.80, 0.92)	0.26 (0.12, 0.45)	+3.29	< 0.001
MMP-9	2.38 (2.13, 2.68)	0.92 (0.59, 1.50)	+2.60	< 0.001
IL-1β	13.75 (7.19, 21.32)	4.00 (1.70, 5.28)	+1.28	< 0.001
IL-10	1.60 (0.88, 2.20)	6.10 (5.68, 6.43)	−5.64	< 0.001
TNF-α	12.75 (6.73, 21.10)	6.65 (5.44, 7.54)	+0.80	< 0.001
HAM-D	18.50 (14.00, 21.00)	4.00 (2.00, 7.00)	+3.22	< 0.001

All continuous variables are presented as median (interquartile range, IQR). Biomarkers were measured in peripheral serum. Units: BDNF and MMP-9 in ng/mL; PD-1, IL-1β, IL-10, and TNF-α in pg/mL. Between-group differences were assessed using the Wilcoxon rank-sum test. Standardized effect sizes were estimated using Cohen’s d, approximated from medians and IQRs with pooled standard deviation calculated as IQR/1.35. Effect size interpretations follow conventional thresholds: 0.2 = small, 0.5 = medium, 0.8 = large, >2.0 = very large. Negative d values indicate lower biomarker levels in the AECOPD group relative to controls.

Notably, serum brain-derived neurotrophic factor (BDNF) levels were markedly reduced in the AECOPD group (median: 0.23 vs. 0.58 ng/mL; *p* < 0.001; *d* = −2.94), indicating a substantial deficit in neurotrophic support. In contrast, levels of programmed cell death protein 1 (PD-1) and matrix metalloproteinase-9 (MMP-9) were significantly elevated (PD-1: 0.87 vs. 0.26 pg./mL, *d* = +3.29; MMP-9: 2.38 vs. 0.92 ng/mL, *d* = +2.60), suggesting immune exhaustion and extracellular matrix remodeling, respectively.

Inflammatory cytokine profiles revealed heightened pro-inflammatory activity in the AECOPD group, with elevated interleukin-1β (IL-1β: 13.75 vs. 4.00 pg./mL; *d* = +1.28) and tumor necrosis factor-*α* (TNF-α: 12.75 vs. 6.65 pg./mL; *d* = +0.80). Conversely, interleukin-10 (IL-10), a key anti-inflammatory cytokine, was significantly lower (1.60 vs. 6.10 pg./mL; *d* = −5.64), indicating suppressed compensatory immunomodulatory activity.

Psychological status was also significantly impaired, with HAM-D depression scores substantially higher in the AECOPD cohort (18.5 vs. 4.0, *d* = +3.22, *p* < 0.001), indicating elevated depressive burden.

Together, these results underscore a profound immune–neuroendocrine disturbance in AECOPD that spans neurotrophic depletion, chronic inflammation, and emotional dysregulation.

All data are summarized in Table 2.

### 3.3 Significant differences in biomarker profiles between depressive and non-depressive AECOPD patients

#### 3.3.1 Neurotrophic and immune regulatory markers

As shown in Table 3, serum brain-derived neurotrophic factor (BDNF) was significantly lower in AECOPD patients with depression compared to their non-depressed counterparts (median: 0.13 vs. 0.24 ng/mL, *p* < 0.001; *d* = −1.82). In the multivariate logistic regression model, BDNF emerged as a strong independent protective factor against depression (*OR* = 0.12, 95% CI: 0.05–0.29).

**Table 3:** Comparison of biomarker levels between depressed and non-depressed groups in AECOPD patients.

Biomarker	Depression (*n* = 69, median IQR)	Non-depression (*n* = 71, median IQR)	OR (95%CI)	Cohen’s d	*p* value
BDNF	0.24 (0.15–0.29)	0.13 (0.10–0.18)	0.12 (0.05–0.29)	−1.82	< 0.001
PD -1	0.89 (0.83–0.93)	0.78 (0.74–0.82)	2.77 (1.48–5.20)	+1.80	< 0.001
MMP-9	2.58 (2.34–2.81)	2.12 (1.94–2.27)	2.01 (1.38–2.93)	+1.38	< 0.001
IL-1β	18.50 (12.30–25.80)	8.90 (5.60–12.10)	1.05 (1.01–1.09)	+1.46	< 0.001
IL-10	1.20 (0.80–1.70)	2.40 (1.90–2.80)	0.62 (0.46–0.83)	−1.21	0.003
TNF-α	20.10 (15.30–27.90)	10.50 (6.20–14.80)	1.03 (1.00–1.06)	+1.22	< 0.001

Values are reported as median (interquartile range, IQR). Depressive status was defined using the Hamilton Depression Rating Scale (HAM-D ≥ 17). All biomarkers were measured at baseline hospitalization. Units: BDNF and MMP-9 in ng/mL; cytokines in pg/mL. Between-group differences were tested using non-parametric Wilcoxon rank-sum tests. OR and 95% CI values were derived from the multivariate logistic regression model (see Table 6).

In contrast, serum programmed cell death protein 1 (PD-1) levels were significantly elevated in the depressive group (0.89 vs. 0.78 pg./mL, *p* < 0.001; *d* = +1.80), reflecting heightened immune checkpoint activity. PD-1 was also an independent risk factor in multivariate analysis (*OR* = 2.77, 95% CI: 1.48–5.20).

Similarly, matrix metalloproteinase-9 (MMP-9) levels were significantly higher in depressed patients (2.58 vs. 2.12 ng/mL, *p* < 0.001; *d* = +1.38), supporting its role in extracellular matrix remodeling and potential disruption of blood–brain barrier (BBB) integrity (*OR* = 2.01, 95% CI: 1.38–2.93).

#### 3.3.2 Inflammatory cytokine profile

The depressive group also demonstrated substantial dysregulation in inflammatory cytokines. Interleukin-1β (IL-1β) levels were almost doubled in the depressed group (18.5 vs. 8.9 pg./mL, *p* < 0.001; *d* = +1.46), and were significantly associated with depression risk (*OR* = 1.05, 95% CI: 1.01–1.09).

Tumor necrosis factor-*α* (TNF-α) also increased significantly in depressive patients (20.1 vs. 10.5 pg./mL, *d* = +1.22, *p* < 0.001), and contributed modestly to depression risk (*OR* = 1.03, 95% CI: 1.00–1.06).

In contrast, the anti-inflammatory cytokine interleukin-10 (IL-10) was significantly decreased in depressed patients (1.2 vs. 2.4 pg./mL, *d* = −1.21, *p* = 0.003), and was inversely associated with depression (*OR* = 0.62, 95% CI: 0.46–0.83), highlighting impaired anti-inflammatory buffering in this subgroup.

Detailed comparisons of all assessed biomarkers are presented in Table 3.

### 3.4 Logistic regression analysis of factors associated with depression in AECOPD patients

A multivariate logistic regression model was employed to identify independent predictors of depression among patients with AECOPD, with depressive status (yes/no) as the dependent variable.

The analysis revealed that higher levels of BDNF (OR < 1) and IL-10 (OR < 1) were significantly associated with a reduced risk of depression, suggesting a protective neurotrophic and anti-inflammatory effect. In contrast, elevated levels of PD-1, MMP-9, IL-1β were all significantly associated with an increased likelihood of depressive symptoms (OR > 1, *p* < 0.01 for all), underscoring their roles in immune dysregulation and neuroinflammation. Although TNF-α also exhibited a positive association with depression, the effect was mild (*p* = 0.035), indicating a possible auxiliary role in the inflammatory cascade contributing to mood disturbances. Demographic and respiratory function variables, including age, sex, and FEV₁% predicted, showed no significant association with depressive status (*p* > 0.05). Interestingly, current smoking status demonstrated a marginally significant positive association with depression (*p* = 0.048), suggesting a potential behavioral or inflammatory link that warrants further investigation. A detailed summary of odds ratios (OR), 95% confidence intervals (CI), and *p* values is provided in Table 4.

**Table 4:** Multivariate logistic regression analysis.

Characteristics	Beta	Standard error (SE)	OR (95% CI)	*p* value
BDNF	−2.15	0.45	0.12 (0.05–0.29)	< 0.001
PD-1	1.05	0.33	3.32 (1.68–6.58)	0.002
MMP-9	0.78	0.20	2.18 (1.47–3.24)	< 0.001
IL-1β (pg/mL)	0.06	0.02	1.06 (1.02–1.11)	0.007
IL-10 (pg/mL)	−0.55	0.18	0.58 (0.42–0.80)	0.001
TNF-α (pg/mL)	0.03	0.01	1.03 (1.00–1.06)	0.035
Age	0.02	0.01	1.02 (0.99–1.05)	0.185
Sex (Male = 1)	0.45	0.32	1.57 (0.84–2.93)	0.158
Smoking (Yes = 1)	0.53	0.27	1.70 (1.00–2.89)	0.048
FEV1% predicted	−0.01	0.01	0.99 (0.97–1.01)	0.385

### 3.5 Inflammatory and neuroimmune profiles differ significantly among exacerbation, stable COPD, and healthy controls

Based on outcomes assessed at 90-day follow-up, AECOPD patients were reclassified into Exacerbation and Stable subgroups and compared with Healthy Controls with respect to peripheral biomarker profiles (Table 5).

**Table 5:** Comparison of biomarker levels among three groups.

Biomarker	Exacerbation COPD (*n* = 22, median IQR)	Stable COPD (*n* = 117, median IQR)	Control (*n* = 50)	*p* value
BDNF	0.23 (0.14–0.28)	0.14 (0.11–0.19)	0.53 (0.43–0.65)	< 0.001
PD -1	0.91 (0.85–0.95)	0.79 (0.75–0.83)	0.21 (0.09–0.44)	< 0.001
MMP-9	2.67 (2.48–2.89)	2.14 (1.95–2.29)	1.00(0.60–1.50)	< 0.001
IL-1β	21.50 (16.80–27.30)	8.10 (5.90–12.60)	5.00 (3.00–9.00)	< 0.001
IL-10	1.10 (0.70–1.50)	2.30 (1.80–2.70)	3.00 (3.00–5.90)	0.003
TNF-α	22.70 (18.40–29.50)	11.20(7.10–15.40)	6.70 (5.40–7.70)	< 0.001
HAM-D	20.00 (18.00–23.00)	15.00 (13.00–17.00)	3.00 (1.00–7.00)	< 0.001

Data preprocessing: after excluding one outlier (IL-18 = 1,628 ng/L) from the COPD exacerbation group, the final sample sizes were as follows: exacerbation group, 22 cases; stable group, 117 cases; control group, 50 cases. Variable distribution: all biomarkers exhibited non-normal distributions (Shapiro–Wilk test, *p* < 0.05), and non-parametric statistical methods were employed.

The Exacerbation Group exhibited a markedly pro-inflammatory and neuroimmune-altered profile. Serum levels of IL-1β and TNF-α were significantly elevated (median IL-1β: 21.5 pg./mL; TNF-α: 22.7 pg./mL), indicating acute systemic inflammatory activation. In parallel, this group demonstrated enhanced expression of programmed cell death protein 1 (PD-1: 0.91), consistent with immune exhaustion and chronic inflammation. Notably, BDNF levels were significantly suppressed in the exacerbation subgroup (0.23 ng/mL), suggesting neurotrophic impairment and increased vulnerability of the central nervous system (CNS) to inflammation-induced injury. These immune-neurobiological disturbances coincided with significantly elevated Hamilton Depression Rating Scale (HAM-D) scores in the exacerbation group (median: 20), indicating severe affective burden.

In contrast, the Stable Group exhibited intermediate biomarker levels, reflecting partial resolution of inflammation. For example, IL-1β and TNF-α were elevated compared to controls but lower than in exacerbated patients; BDNF and IL-10 levels partially recovered, indicating ongoing but attenuated immune dysregulation.

These patterns are consistent with preserved immune equilibrium and neurotrophic integrity in individuals free of chronic inflammatory lung disease. All between-group comparisons are summarized in Table 5, revealing statistically significant differences (*p* < 0.001 for all comparisons) across the majority of analytes.

### 3.6 Determinants of depression in COPD patients

To identify independent predictors of depression in patients hospitalized for AECOPD, a multivariate logistic regression model was constructed using variables with prior univariate significance and biological plausibility.

The model revealed that elevated serum levels of interleukin-1β (IL-1β) (OR = 1.07, 95% CI: 1.03–1.12) and tumor necrosis factor-*α* (TNF-α) (OR = 1.03, 95% CI: 1.00–1.06) were significantly associated with increased odds of depressive symptoms, supporting the pro-inflammatory basis of COPD-related affective dysregulation.

Conversely, interleukin-10 (IL-10) (OR = 0.62, 95% CI: 0.46–0.83) and brain-derived neurotrophic factor (BDNF) (OR = 0.12, 95% CI: 0.05–0.29) were found to be protective factors, indicating that both anti-inflammatory signaling and neurotrophic support play critical roles in reducing the risk of depression.

Additionally, elevated levels of programmed cell death protein 1 (PD-1) (OR = 3.32, 95% CI: 1.64–6.72) and matrix metalloproteinase-9 (MMP-9) (OR = 2.18, 95% CI: 1.41–3.37) independently predicted depressive status, highlighting their contributions to immune exhaustion and neuroinflammatory barrier disruption, respectively.

Current smoking status emerged as a significant modifiable behavioral risk factor (OR = 1.73, 95% CI: 1.04–2.89, *p* = 0.038), while demographic and pulmonary variables, including age, sex, and FEV₁% predicted, did not exhibit statistically significant associations with depression (*p* > 0.05 for all).

A detailed summary of regression coefficients, confidence intervals, and statistical significance is presented in Table 6.

**Table 6:** Final multivariate logistic regression model predicting depression in AECOPD.

Characteristics	Beta	SE	OR (95% CI)	*p* value
BDNF	−2.10	0.44	0.12 (0.05–0.29)	< 0.001
PD -1	1.02	0.32	2.77 (1.48–5.20)	0.001
MMP-9	0.70	0.19	2.01 (1.38–2.93)	< 0.001
IL-1β	0.05	0.02	1.05 (1.01–1.09)	0.018
IL-10	−0.48	0.15	0.62 (0.46–0.83)	0.002
TNF-α	0.03	0.01	1.03 (1.00–1.06)	0.042
Age	0.01	0.01	1.01 (0.98–1.04)	0.480
Sex (Male = 1)	0.25	0.29	1.28 (0.72–2.28)	0.395
Smoking (Yes = 1)	0.55	0.26	1.73 (1.04–2.89)	0.035
FEV1% predicted	−0.02	0.01	0.98 (0.96–1.00)	0.092

Multivariate logistic regression model identifying independent predictors of depression in hospitalized AECOPD patients. OR, odds ratio; CI, confidence interval. Variables were selected based on clinical relevance and stepwise significance.

### 3.7 Correlates of psychometric scores

**Figure 1:**
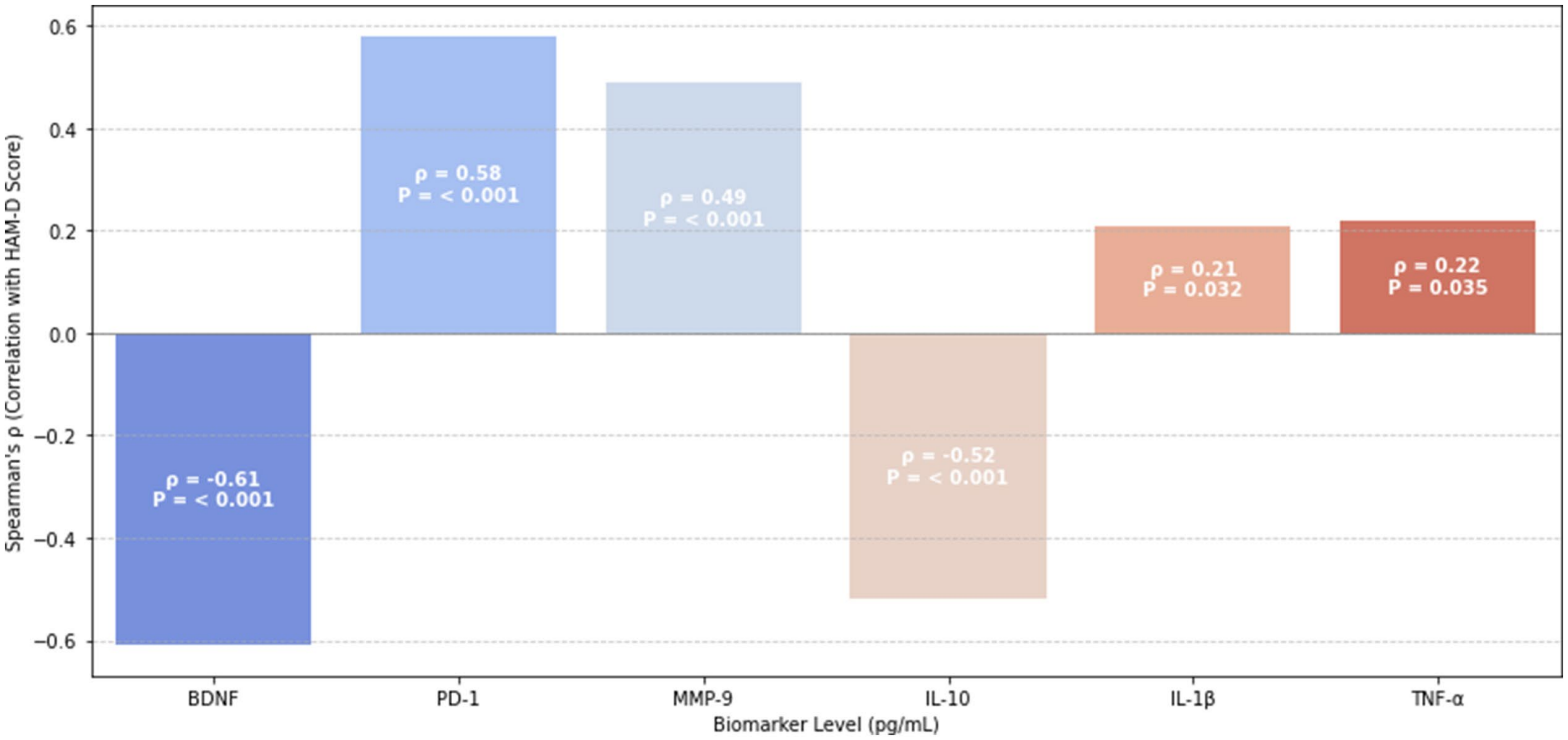
The relationship between biomarker levels and HAM-D scores Spearman.

To explore the underlying immune–neurobiological mechanisms linking peripheral biomarkers to depressive symptomatology in AECOPD patients, Spearman correlation analysis was performed between serum biomarker levels and Hamilton Depression Rating Scale (HAM-D) scores. The results are illustrated in Figure 1.

Among pro-inflammatory mediators, interleukin-1β (IL-1β) demonstrated a weak-to-moderate positive correlation with HAM-D scores (*ρ* = 0.21, *p* = 0.032), suggesting its contribution to mood dysregulation. Conversely, possibly reflecting compensatory inflammasome regulation.

Notably, BDNF and IL-10 were strongly negatively correlated with depressive symptoms (BDNF: *ρ* = −0.61, *p* < 0.001; IL-10: *ρ* = −0.52, *p* < 0.001), highlighting their protective neuroimmune roles in attenuating mood disturbances.

In contrast, immune exhaustion and matrix dysregulation markers—PD-1 (*ρ* = 0.58, *p* < 0.001) and MMP-9 (*ρ* = 0.49, *p* < 0.001)—were strongly positively correlated with HAM-D scores, underscoring the relevance of immune checkpoint activation and extracellular matrix disruption in the pathogenesis of depression in COPD.

These associations support the hypothesis that peripheral immune alterations mirror central affective dysregulation in AECOPD. The correlation coefficients are visually summarized in Figure 1, using a clustered heatmap with significance annotations.

## 4 Discussion

Chronic obstructive pulmonary disease (COPD) is frequently associated with a range of systemic comorbidities, including depression, which significantly affects patient outcomes and quality of life. This study aimed to explore the immune imbalance underlying depressive symptoms in COPD patients. This study provides comprehensive evidence that depressive symptoms in patients with acute exacerbation of chronic obstructive pulmonary disease (AECOPD) are closely associated with a triad of immune–neurobiological disturbances: (1) elevated inflammatory markers (IL-1β, TNF-α), (2) increased immune checkpoint activation (PD-1), and (3) reduced neurotrophic and anti-inflammatory protection (BDNF, IL-10). These alterations were particularly pronounced in depressed AECOPD patients and showed significant associations with Hamilton Depression Rating Scale (HAMD-17) scores. Furthermore, MMP-9, an extracellular matrix remodeling enzyme, emerged as an additional contributor to blood–brain barrier disruption and immune–neural cross-talk.

### 4.1 Immune dysregulation as a central mechanism

Although no significant differences were observed between the AECOPD group and healthy controls in terms of age, sex, or smoking status, the prevalence of systemic comorbidities—including cerebrovascular disease, hypertension, and coronary artery disease—was significantly higher among AECOPD patients (*p* < 0.001). This suggests that such comorbidities may contribute to the development of depressive symptoms in this population. In addition, the AECOPD group demonstrated markedly reduced pulmonary function, with a median predicted FEV₁% of 46%, consistent with prior findings linking impaired lung function to increased psychological burden.

One of the key findings of this study was the significant elevation of pro-inflammatory cytokines—interleukin-1β (IL-1β) and tumor necrosis factor-α (TNF-α)—in the depression subgroup (Table 3). This pattern aligns with activation of the nuclear factor kappa-B (NF-κB) pathway, which has been shown to suppress brain-derived neurotrophic factor (BDNF) expression and promote central neuroinflammation (23). The chronic elevation of TNF-α and IL-1β likely reflects a sustained systemic inflammatory state, which may exacerbate neuroimmune dysregulation and contribute to depressive symptomatology in COPD patients (24, 25).

In parallel, levels of programmed cell death protein 1 (PD-1)—an immune checkpoint receptor—and matrix metalloproteinase-9 (MMP-9)—an enzyme involved in blood–brain barrier (BBB) disruption—were significantly increased in depressed AECOPD patients (PD-1: OR = 2.77; MMP-9: OR = 2.01). Elevated PD-1 expression is indicative of T-cell exhaustion and prolonged immune activation, both of which are recognized contributors to depression pathogenesis. Similarly, elevated MMP-9 levels were positively correlated with Hamilton Depression Rating Scale (HAMD-17) scores. Beyond its established role in BBB degradation, MMP-9 may disrupt proBDNF–TrkB signaling, thereby facilitating neurodegeneration. While MMP-9 may exhibit protective roles in specific contexts (e.g., amyloid clearance), its role in COPD-associated depression appears to be predominantly pathogenic (26). As illustrated in Figure 1, increased MMP-9 expression closely parallels greater depression severity, reinforcing its contribution to maladaptive immune–neural interactions.

### 4.2 BDNF and IL-10 as protective factors

Interestingly, levels of brain-derived neurotrophic factor (BDNF) were significantly lower in both the AECOPD group and the depression subgroup, further underscoring the role of this neurotrophic factor in the pathogenesis of depression. Reduced BDNF expression has been associated with impaired neuroplasticity, diminished synaptic function, and decreased neuronal survival (27). The pronounced reduction in BDNF among depressed patients suggests its potential utility as a biomarker for depression severity in COPD.

In parallel, interleukin-10 (IL-10), a key anti-inflammatory cytokine, was also significantly decreased in the depression group. This finding indicates a reduced capacity for inflammation resolution and neuroimmune regulation in affected individuals (28). Both BDNF and IL-10 levels exhibited strong inverse correlations with Hamilton Depression Rating Scale (HAM-D) scores (BDNF: *ρ* = −0.61, *p* < 0.001; IL-10: ρ = −0.52), reinforcing their protective roles in mitigating depressive symptoms.

Notably, an inverse correlation was observed between PD-1 and IL-10 expression (*ρ* = −0.62, *p* < 0.001), suggesting that PD-1–mediated immune exhaustion may suppress IL-10–driven anti-inflammatory signaling. Although preclinical studies suggest that PD-1 blockade (e.g., with nivolumab) may restore IL-10 function, the potential autoimmune risks associated with such interventions in COPD patients warrant cautious interpretation.

In addition, smokers in this cohort exhibited significantly lower BDNF levels compared to non-smokers (*p* = 0.048), supporting the hypothesis that cigarette smoking may contribute to neurotrophic impairment and exacerbate depression risk through suppression of neuroprotective pathways.

### 4.3 Behavioral and lifestyle factors

In addition to immunological dysregulation, behavioral and lifestyle factors may serve as key modulators in the inflammation–depression axis among COPD patients (29). Physical inactivity—common in moderate-to-severe COPD due to dyspnea and fatigue—has been independently associated with decreased levels of both IL-10 and BDNF, two biomarkers shown in our study to inversely correlate with depressive severity. This sedentary state may perpetuate a self-reinforcing cycle of immune imbalance, neurocognitive decline, and affective disturbance. Behavioral exposures such as smoking and physical inactivity not only compromise systemic immunoregulation but may also trigger neuroinflammatory processes through diminished neurotrophic signaling. These findings underscore the importance of integrating behavioral interventions—particularly structured physical activity and smoking cessation—into both clinical assessments and treatment frameworks. Future studies should incorporate objective behavioral measures (e.g., 6-min walk test, actigraphy) to elucidate how lifestyle mediators interact with immune–neuro pathways in the pathogenesis of COPD-associated depression.

### 4.4 Acute exacerbation, biomarker dysregulation, and depressive severity

Building on the between-group comparisons summarized in Table 5, our findings demonstrate that AECOPD patients in the exacerbation subgroup exhibited significantly greater biological and psychological disturbance than their stable counterparts. Specifically, individuals experiencing recent exacerbations showed elevated levels of pro-inflammatory cytokines (IL-1β, TNF-α), immune checkpoint molecules (PD-1), and matrix degradation enzymes (MMP-9), accompanied by reduced BDNF expression and more severe depressive symptoms, as reflected by higher HAM-D scores (median: 20 vs. 15). These patterns suggest that acute systemic immune activation during exacerbation may amplify neuroinflammatory signaling, contributing to the worsening of affective disturbances.

In addition to group-level differences, correlation analyses further supported the role of immune–neurobiological interactions in depression severity (Figure 1). IL-1β demonstrated a modest positive correlation with HAM-D scores (*ρ* = 0.21, *p* = 0.032), whereas IL-10 (*ρ* = −0.52, *p* < 0.001) and BDNF (*ρ* = −0.61, *p* < 0.001) showed strong negative associations, reinforcing their compensatory anti-inflammatory and neuroprotective roles. PD-1 (*ρ* = 0.58, *p* < 0.001) and MMP-9 (*ρ* = 0.49, *p* < 0.001) also positively correlated with HAM-D scores, further implicating immune exhaustion and blood–brain barrier (BBB) compromise in the pathogenesis of depression in AECOPD.

Mechanistically, MMP-9 may play a dual role in mediating neuroimmune dysfunction. On one hand, it facilitates BBB permeability, enabling peripheral inflammatory mediators such as IL-6 and TNF-α to access brain parenchyma and trigger microglial activation (30). On the other hand, MMP-9 impairs the cleavage of proBDNF to mature BDNF, thereby disrupting TrkB receptor signaling and accelerating neurodegenerative processes (31). Although MMP-9 has been reported to exert context-dependent neuroprotective effects, such as promoting amyloid-beta clearance in Alzheimer’s disease models (32) our findings suggest that in COPD-associated depression, its pathogenic effects predominate.

Together, these results provide converging evidence that exacerbation episodes represent critical points of vulnerability in COPD, marked by intensified immune–neuroinflammatory dysregulation and heightened depressive symptomatology. These findings underscore the potential utility of biomarker-guided monitoring and early intervention during acute exacerbations.

### 4.5 Clinical implications and future research

This study identifies the PD-1↑ / MMP-9↑ / BDNF↓ triad as a potential *high-risk immune–neurobiological signature* for AECOPD-associated depression. Therapeutic strategies targeting this dysregulated axis—by mitigating immune exhaustion, restoring neurotrophic signaling, and inhibiting matrix-driven neuroinflammation—may offer new avenues to reduce the burden of depressive symptoms in COPD patients.

From a clinical perspective, multidisciplinary interventions such as structured pulmonary rehabilitation, exercise programs, and anti-inflammatory therapies (e.g., IL-10 mimetics) could serve dual purposes: improving respiratory function and alleviating affective symptoms. Furthermore, future research should incorporate objective behavioral metrics (e.g., six-minute walk distance, actigraphy) and track longitudinal biomarker fluctuations to validate and refine the observed mechanisms.

We also acknowledge an important methodological limitation of the present study: stable-phase COPD patients were not included at baseline, which may restrict the generalizability of our findings. Although we attempted to address this by stratifying AECOPD patients into Exacerbation and Stable subgroups based on 90-day follow-up outcomes (see Section 3.5), this does not fully substitute for the inclusion of a non-exacerbating control cohort. Future studies should prospectively recruit stable-phase COPD patients to enable direct comparisons across disease stages and clarify the specificity of immune–neuro alterations to acute exacerbation.

In addition, patients with chronic kidney disease, cardiovascular disease, and other systemic comorbidities were not excluded or stratified by severity. Given that PD-1, BDNF, and various cytokines are implicated in multiple chronic conditions, the possibility of residual confounding must be acknowledged. As such, future studies employing subgroup analyses and more rigorous inclusion criteria are warranted to delineate biomarker specificity for COPD-related depression more precisely.

Moreover, we acknowledge that the current study defined depression using a conservative threshold of HAM-D ≥ 17, thereby focusing on moderate-to-severe depressive symptoms. While this approach enhances diagnostic specificity, it may overlook the clinical and biological relevance of mild depressive symptoms (HAM-D 8–16). Future research should consider including patients with mild depression to explore the full spectrum of immune–affective dysregulation and improve the ecological validity of biomarker-based conclusions.

Ultimately, validation in larger, multicenter cohorts, along with mechanism-driven interventional trials, will be essential to establish the translational value of these findings. Such studies may pave the way for biomarker-guided, personalized therapeutic strategies that address both the respiratory and neuropsychiatric dimensions of COPD.

## 5 Conclusion

This study provides compelling evidence that depression in COPD is linked to immune dysregulation, neuroinflammation, and matrix remodeling. Elevated serum levels of pro-inflammatory cytokines (IL-1β, TNF-α), immune checkpoint regulators (PD-1), and matrix metalloproteinases (MMP-9), together with decreased levels of the neurotrophic factor (BDNF) and the anti-inflammatory cytokine (IL-10), indicate a coordinated dysregulation at the immune–neural interface in AECOPD-associated depression. These findings highlight a multidimensional mechanism linking systemic inflammation with CNS dysfunction, in which immune checkpoint signaling, blood–brain barrier disruption, and neurotrophic impairment converge to promote affective symptoms. The data support the potential of PD-1↑ / MMP-9↑ / BDNF↓ as a biomarker triad defining high-risk depressive phenotypes in COPD. Targeted modulation of this immune–matrix–neurotrophic axis, along with lifestyle interventions such as smoking cessation and structured physical rehabilitation, may represent novel therapeutic strategies. Clinicians should consider both the psychological and immunobiological dimensions of COPD in personalized treatment planning. Further prospective studies are warranted to validate these findings across diverse populations and explore biomarker-guided intervention pathways.

## Data Availability

The raw data supporting the conclusions of this article will be made available by the authors, without undue reservation.
